# Mortality Predictors in Renal Transplant Recipients with Severe Sepsis and Septic Shock

**DOI:** 10.1371/journal.pone.0111610

**Published:** 2014-11-04

**Authors:** Mônica Andrade de Carvalho, Flávio Geraldo Rezende Freitas, Hélio Tedesco Silva Junior, Antônio Toneti Bafi, Flávia Ribeiro Machado, José Osmar Medina Pestana

**Affiliations:** 1 Unidade de Transplante, Disciplina de Nefrologia, Universidade Federal de São Paulo, São Paulo, SP, Brazil; 2 Disciplina de Anestesiologia, Dor e Terapia Intensiva. Universidade Federal de São Paulo, São Paulo, SP, Brazil; University of California, San Diego, United States of America

## Abstract

**Introduction:**

The growing number of renal transplant recipients in a sustained immunosuppressive state is a factor that can contribute to increased incidence of sepsis. However, relatively little is known about sepsis in this population. The aim of this single-center study was to evaluate the factors associated with hospital mortality in renal transplant patients admitted to the intensive care unit (ICU) with severe sepsis and septic shock.

**Methods:**

Patient demographics and transplant-related and ICU stay data were retrospectively collected. Multiple logistic regression was conducted to identify the independent risk factors associated with hospital mortality.

**Results:**

A total of 190 patients were enrolled, 64.2% of whom received kidneys from deceased donors. The mean patient age was 51±13 years (males, 115 [60.5%]), and the median APACHE II was 20 (16–23). The majority of patients developed sepsis late after the renal transplantation (2.1 [0.6–2.3] years). The lung was the most common infection site (59.5%). Upon ICU admission, 16.4% of the patients had ≤1 systemic inflammatory response syndrome criteria. Among the patients, 61.5% presented with ≥2 organ failures at admission, and 27.9% experienced septic shock within the first 24 hours of ICU admission. The overall hospital mortality rate was 38.4%. In the multivariate analysis, the independent determinants of hospital mortality were male gender (OR = 5.9; 95% CI, 1.7–19.6; p = 0.004), delta SOFA 24 h (OR = 1.7; 95% CI, 1.2–2.3; p = 0.001), mechanical ventilation (OR = 30; 95% CI, 8.8–102.2; p<0.0001), hematologic dysfunction (OR = 6.8; 95% CI, 2.0–22.6; p = 0.002), admission from the ward (OR = 3.4; 95% CI, 1.2–9.7; p = 0.02) and acute kidney injury stage 3 (OR = 5.7; 95% CI,1.9–16.6; p = 0.002).

**Conclusions:**

Hospital mortality in renal transplant patients with severe sepsis and septic shock was associated with male gender, admission from the wards, worse SOFA scores on the first day and the presence of hematologic dysfunction, mechanical ventilation or advanced graft dysfunction.

## Introduction

Sepsis is the leading cause of death in non-cardiac intensive care units, although there is some evidence of a decline in mortality rates, at least in developed countries [1]–[4]. The scenario in emerging and limited-resources countries seems to be different with higher reported rates [5], [6], although low mortality rates has also been reported [7]. The incidence of sepsis is increasing over the past years and the growing number of patients living with solid organ transplants is a factor that contributes to this finding [2]–[4], [8].

The most common solid organ transplant procedure worldwide is the renal transplantation. It is the treatment of choice for end-stage renal disease. Compared with chronic dialysis, renal transplantation is cost-effective, offers improved quality of life and confers a progressive survival benefit [9], [10]. The overall survival rate of kidney grafts has improved consistently during the past decades [11]. Moreover, the number of adult candidates on the waiting lists with kidney failure continues to increase [12]. Therefore, more renal transplant recipients with functioning grafts will be exposed to pathogens while in a sustained immunosuppressive state.

Because of immunosuppression, infection frequently occurs after kidney transplantation and greatly impacts patient morbidity and mortality. This explains why infection is the second leading cause of death in renal transplant recipients, following cardiovascular diseases [13]. The importance of infection as cause of death is higher in underdeveloped countries [14], [15]. Surprisingly, relatively little is known about severe sepsis in this growing population. The aim of this study was to describe the characteristics of severe sepsis and septic shock in renal transplant patients who are admitted to the intensive care unit (ICU) and to evaluate the factors associated with hospital mortality.

## Materials and Methods

This single center, retrospective, observational study was performed at a kidney transplant center in Brazil [16]. The institutional ethics committee approved the study and waived the informed consent requirement (Comitê de Ética em Pesquisa – Universidade Federal de São Paulo, reference number: 1736–10). All consecutive adult renal transplant recipients (older than 18 years) diagnosed with severe sepsis or septic shock who were admitted to our 12-bed ICU from June 1, 2010 to December 31, 2011 were included. We excluded pregnant patients, patients who underwent kidney-pancreas transplantation, and patients with “do not resuscitate” orders. All patients were included only in their first episode of sepsis.

Data were retrospectively collected through medical records by a single author (MAC). We recorded the following data: patient demographics, comorbid chronic illnesses, severe sepsis characteristics and the severity scores Sequential Organ Failure Assessment (SOFA) and Acute Physiology and Chronic Health Evaluation II (APACHE II). We also collected data on the initial treatment, life support and fluid balance as well as pre-transplant, peritransplant and post-transplant variables. We assessed adequacy of treatment according to the compliance to the 6-hours Surviving Sepsis Campaign bundle available during the study period [17], which are similar to the recent published 3-hour and 6-hour bundles of the 2012 revised guidelines [18]. All transplant patients in our hospital are under continuous surveillance. Thus, the hospital database has all information about outpatient's visits, hospital readmissions or death in other institutions. Thus, we collected not only the hospital mortality during the septic episode but also the one-year survival. The database was reviewed by two authors (FGRF and FRM). In cases of inconsistency, the sources documents were verified, and the data were corrected. Data were anonymized and de-identified prior to data analysis.

Severe sepsis was defined as a documented or presumed infection plus at least one organ failure secondary to infection. We did not use the systemic inflammatory response syndrome (SIRS) criteria, as depressed febrile response and diminished leukocytosis are frequently seen in solid-organ recipients [19]. Septic shock was defined as volume-refractory hypotension with the need for vasopressor. Organ dysfunction was diagnosed when one of the following factors was present: hypotension with systolic blood pressure <90 mmHg or mean arterial blood pressure <65 mmHg (cardiovascular); arterial oxygen partial pressure/oxygen inspiratory fraction (PaO_2_/FiO_2_) ratio ≤300 (respiratory); a bilirubin level > twice the reference value (hepatic); a lactate level ≥1.5 times the reference value and a base deficit >5 (metabolic); an international normalized ratio (INR) >1.5 or a platelet count <100,000/mL (hematologic) and altered level of consciousness (neurologic). To define renal dysfunction, we used increased serum creatinine > twice the baseline value. This cutoff was arbitrary chosen because of the lack of agreement on the definition of acute kidney injury (AKI) in this population. In parallel, we also used the definition recommended by Kidney Disease: Improving Global Outcomes (KDIGO) [20] to stage AKI during the ICU stay, without considering urine output.

The time to the sepsis diagnosis was defined as the number of hours elapsed between the onset of the first organ dysfunction and the recognition and management of sepsis by the healthcare provider, as described elsewhere [21]. The severe sepsis and septic shock treatment was analyzed based on compliance with the initial care bundle (within the first 6 hrs of presentation) [22].

### Statistical methods

The categorical variables are described as percentages, and the continuous variables are described as measures of central tendency and dispersion, according to distribution, as assessed by the Kolmogorov-Smirnov test. We compare hospital survivors and non-survivor using the two-tailed t-test, Mann-Whitney U-test, chi-squared test, and Fisher's exact test, as appropriate. Multiple logistic regression was conducted to identify the independent risk factors associated with hospital mortality, including all variables with a p value <0.10 in the univariate analysis (using a stepwise forward regression model). The time until the sepsis diagnosis was categorized using the best cutoff value in the receiver operating characteristic (ROC) curve for mortality (≥170 vs. <170 min). The number of organ dysfunctions (≥2 vs. <2) and the KDIGO classification (stage 3 vs. stage <3) of acute kidney injury during ICU stay were also categorized. All variables were checked for confounding and collinearity. The model calibration was assessed using the Hosmer-Lemeshow test, which was considered to be appropriate if p>0.10. We did not include the variables with missing data >10%, as the lack of data would result in serious inconsistencies. The patients were followed for one year, and a mortality curve was generated using the Kaplan-Meier methodology. A p value <0.05 was considered to be significant. Data were analyzed using SPSS 19.0 for Windows (SPSS, Chicago, IL, USA).

## Results

**Figure 1:**
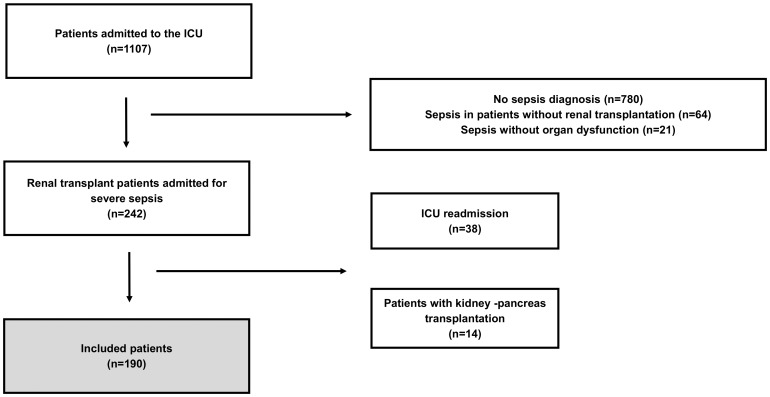
Study flowchart. ICU: intensive care unit.

During the study period, 1107 patients were admitted to the ICU, 242 (21.9%) of whom were renal transplant patients who were admitted for severe sepsis. Of these patients, 190 were enrolled, as shown in Figure 1.

The patients' characteristics and transplant variables are summarized in Table 1. The leading causes of end-stage renal disease were glomerulonephritis (26.3%), diabetes mellitus (18.9%) and hypertension (14.7%), although most patients (35.2%) did not have an identifiable cause. The majority of kidneys transplanted were from deceased donors (64.2%). All patients had immunosuppression suspended at ICU admission and used hydrocortisone (50 mg every six hours). The majority of the patients developed sepsis late after the renal transplantation (2.1 years; range, 0.6–2.3 years). Fifty-five patients (28.9%) had histories of acute rejection that occurred at a median of 312 days (range, 130–776 days) before the ICU admission. The univariate analysis showed delayed graft function, and expanded criteria donor kidneys were associated with hospital mortality. No other clinical characteristic related to the transplant was significantly different between the survivors and non-survivors.

**Table 1:** Patient characteristics and transplant variables.

	All Patients (n = 190)	Survivors (n = 117)	Non survivors (n = 73)	p value
Age (years)	51±13	50±13	52±13	0.300
Male gender	115 (60.5)	61 (52.1)	54 (73.9)	**0.002**
Body mass index (kg/m^2^)	24±5	25±5	23±4	**0.003**
Comorbidities				
Hypertension	152 (80.0)	99 (84.6)	53 (72.6)	**0.040**
Diabetes mellitus	61 (32.1)	39 (33.3)	22 (30.1)	0.600
CAD	35 (18.4)	24 (20.5)	11 (15.0)	0.300
Stroke	8 (4.2)	6 (5.1)	2 (2.7)	0.700
CHF	5 (2.6)	3 (2.5)	2 (2.7)	1.000
Hepatitis C	13 (6.8)	7 (5.9)	6 (8.2)	0.500
Hepatitis B	6 (3.1)	3 (2.5)	3 (4.1)	0.600
COPD	6 (3.1)	1 (0.8)	5 (6.8)	**0.030**
ESRD etiology				0.220
Undetermined	67 (35.2)	41 (35.0)	26 (35.6)	
Glomerulonephritis	50 (26.3)	33 (28.2)	17 (23.2)	
Diabetes mellitus	36 (18.9)	18 (15.3)	18 (24.6)	
Hypertension	28 (14.7)	21 (17.9)	7 (9.5)	
Urologic disease	9 (4.7)	4 (3.4)	5 (6.8)	
Dialysis modality before transplant				0.480
Preemptive	8 (4.2)	7 (5.9)	1 (1.3)	
Hemodialysis	153 (80.5)	91 (77.7)	62 (84.9)	
Peritoneal	21 (11.0)	14 (11.9)	7 (9.5)	
Hemodialysis/peritoneal	8 (4.2)	5 (4.2)	3 (4.1)	
Time of dialysis (months)	34 (18–60)	32 (18–60)	36 (24–68)	0.170
Donor type				0.190
Deceased	122 (64.2)	71 (60.6)	51 (69.8)	
Living	68 (35.8)	46 (39.4)	22 (30.2)	
Donor gender ^a^				0.330
Female	71 (42.0)	43 (40.6)	28 (44.5)	
Male	98 (58.0)	63 (59.4)	35 (55.5)	
Deceased donor ^b^				
Cause of death ^c^				0.930
Traumatic brain injury	33 (28.0)	21 (30.0)	12 (25.0)	
Subarachnoid hemorrhage	20 (16.9)	12 (17.1)	8 (16.6)	
Stroke	56 (47.5)	32 (45.7)	24 (50.0)	
Others	9 (7.6)	5 (7.2)	4 (8.4)	
Panel reactive antibodies ^d^				0.660
0–50%	93 (84.5)	55 (83.3)	38 (86.3)	
>51%	17 (15.5)	11 (16.7)	6 (13.6)	
Final creatinine ^e^				0.210
<1.5 mg/dL	31 (32.6)	21 (35.5)	10 (27.7)	
≥1.5 mg/dL	64 (67.4)	38 (64.5)	26 (72.3)	
Cold ischemia time (hours) ^f^	23 (20–27)	23 (20–28)	22 (20–27)	0.630
Expanded criteria donor	31 (26.3)	13 (18.6)	18 (37.5)	**0.020**
Delayed graft function	82 (43.3)	44 (37.6)	38 (52.7)	**0.040**
Thymoglobulin use ^g^	54 (28.5)	34 (29.0)	20 (27.7)	0.870
CMV disease treated	68 (35.9)	41 (35.0)	27 (37.5)	0.750
Current immunosuppression ^h^				0.460
TAC+PRED+AZA	31 (16.3)	16 (13.6)	15 (20.5)	
TAC+PRED+MF	70 (36.8)	48 (41.0)	22 (30.1)	
CSA+PRED+AZA	17 (8.9)	12 (10.2)	5 (6.8)	
CSA+PRED+MF	7 (3.6)	4 (3.4)	3 (4.1)	
TAC/CSA+PRED+EVR/SRL	4 (2.1)	3 (2.5)	1 (1.7)	
SRL/EVR+PRED+MF	9 (4.7)	4 (3.4)	5 (6.8)	
Others	51 (26.8)	30 (25.6)	21 (28.7)	
Time between transplant and sepsis (years)	2.1 (0.6–7.2)	2.3 (0.6–7.8)	1.6 (0.6–7.0)	0.600
Acute rejection	55 (28.9)	34 (29.0)	21 (28.7)	0.960
Time rejection-sepsis (days) ^i^	312 (130–776)	331(115–817)	282 (152–849)	0.900

CAD coronary artery disease, CHF: congestive heart failure, COPD: chronic obstructive pulmonary disease, ESRD: end-stage renal disease, CMV: *cytomegalovirus*, TAC: tacrolimus, PRED: prednisone, AZA: azathioprine, MF: mycophenolate, CSA: cyclosporine, EVR: everolimus, SRL sirolimus.

a) 21 missing data,

b) 122 deceased donors,

c) 4 missing data,

d) 12 missing data,

e) final creatinine refers to the donors' last serum creatinine level, 27 missing data,

f) 3 missing data,

g) patients who used thymoglobulin for treating rejection and/or induction in transplantation,

h) 1 missing data and i) time between the occurrence of rejection and sepsis (total of patients with rejection, 55 patients, 3 patients among the survivors and 6 among the non-survivors were excluded for missing data). The results are expressed as number (%) or median (IQR, 25%–75%) or mean ± standard deviation. Chi-squared test, Mann-Whitney U-test, and Student's t-test (univariate analysis).

The lung was the most common site of infection (59.5%), followed by the urinary tract (16.8%) and abdomen (9.5%) (Table 2). We isolated the etiologic agents in the majority of the patients (57%). Most of these agents were bacteria (Gram-negative, 45.4%; Gram-positive: 20.4%). The other relevant agents were *Mycobacterium tuberculosis* (3.7%), Cytomegalovirus (3.7%) and fungi (24%), including *Pneumocystis jirovecii* (8.3%) (Table 3).

**Table 2:** Severe sepsis characteristics and treatment.

	All patients (n = 190)	Survivors (n = 117)	Non-survivors (n = 73)	p value
Site of infection				**0.006**
Respiratory	113 (59.5)	66 (56.4)	47 (64.3)	
Urinary	32 (16.8)	28 (23.9)	4 (5.4)	
Abdominal	18 (9.5	8 (6.8)	10 (13.7)	
Others	27 (14.2)	15 (12.8)	12 (16.4)	
SIRS criteria				
Tachypnea	142 (74.7)	84 (71.7)	58 (79.4)	0.230
Tachycardia	129 (67.9)	80 (68.3)	49 (67.1)	0.850
Leukocytosis	50 (26.3)	28 (23.9)	22 (30.1)	0.340
Leukopenia	31 (16.3)	16 (13.6)	15 (20.5)	0.210
Fever	46 (24.2)	32 (27.3)	14 (19.1)	0.200
Hypothermia	12 (6.3)	7 (5.9)	5 (6.8)	1.000
Organ failures				
Respiratory	84 (44.2)	43 (36.7)	41 (56.1)	**0.008**
Cardiovascular	78 (41.1)	49(41.8)	29 (39.7)	0.760
Renal	77 (40.5)	51 (43.5)	26 (35.6)	0.270
Hematologic	64 (33.9)	30 (25.6)	34 (46.6)	0.030
Neurologic	50 (26.3)	26 (22.2)	24 (32.8)	0.100
Metabolic	13 (7.9)	5 (4.8)	8 (12.9)	0.070
Hepatic	9 (4.7)	6 (5.1)	3 (4.1)	1.000
Admission				**<0.0001**
Emergency	110 (57.9)	83 (70.9)	27 (36.9)	
Ward	80 (42.1)	34 (29.0)	46 (63.0)	
Number of organs dysfunctions (≥2)	117 (61.5)	65 (55.5)	52 (71.2)	0.030
Glycemia (mg/dl)^a^	149 (121–194)	151 (121–195)	141 (119–193)	0.360
Time to sepsis diagnosis (hours)	2.5 (1.1–5.2)	2 (0.9–4.2)	3.5 (1.5–6.3)	**<0.001**
Time to antibiotics (minutes)	55 (30–120)	60 (30–120)	45 (20–80)	**<0.001**
Duration of ICU stay (days)	6 (3–13)	6 (3–11)	7 (3–16)	0.130
Duration of hospital stay (days)	20 (12–35)	21 (14–38)	15 (8–31)	0.010
Compliance to severe sepsis bundle				
Measure lactate	164 (86.3)	103 (88.0)	61 (83.5)	0.300
Broad-spectrum antibiotics	173 (91.0)	107 (91.5)	66 (90.4)	0.800
Blood cultures before antibiotics	151 (79.5)	93 (79.4)	58 (79.5)	0.990
Fluid resuscitation ^b^	54 (62.3)	39 (75)	15 (44.1)	**0.004**
CVP >8 mm Hg ^c^	6 (15.8)	2 (11.1)	4 (22.2)	0.370
ScvO2 >70% ^c^	14 (36.8)	7 (38.9)	7 (38.9)	1.000
Initial care bundle	74 (39.0)	45 (38.5)	29 (39.7)	0.800

SIRS: systemic inflammatory response syndrome. ICU: intensive care unit. CVP: central venous pressure, ScvO_2_: central venous oxygen saturation.

a) median glycemia during the first 24 h of sepsis,

b) indication to administer 20 ml/kg crystalloid for hypotension or lactate ≥36 mg/dl (n = 86),

c) indication to measure CVP or measure ScvO_2_ (n = 38). The results are expressed as number (%) or median (IQR: 25%–75%). Chi-squared test and Mann-Whitney U-test (univariate analysis).

**Table 3:** Frequencies of infectious agents identified.

	Frequency, n (%)
Gram-negative	**49 (45.4)**
*Escherichia coli*	16 (15.0)
*Klebsiella pneumonia*	13 (12.0)
*Pseudomonas aeruginosa*	8 (7.4)
*Acinetobacter baumanii*	6 (5.5)
*Enterobacter sp*	3 (2.7)
*Proteus mirabilis*	2 (1.8)
*Citrobacter sp*	1 (0.9)
Gram-positive	**22 (20.4)**
*Staphylococcus aureus*	10 (9.2)
*Enterococcus sp*	7 (6.5)
*Staphylococcus epidermidis*	3 (2.7)
*Streptococcus pneumoniae*	1 (0.9)
*Streptococcus viridans*	1 (0.9)
Fungi	**26 (24.0)**
*Candida albicans*	10 (9.2)
*Pneumocystis jiroveci*	9 (8.3)
*Cryptococcus*	2 (1.8)
*Histoplasma capsulatum*	3 (2.7)
*Cândida sp*	2 (1.8)
Others	**11 (10.2)**
*Mycobacterium turbeculosis*	4 (3.7)
*Cytomegalovirus*	4 (3.7)
*Listeria monocytogenes*	1 (0.9)
*Neisseria meningitidis*	1 (0.9)
*Salmonella sp*	1 (0.9)

**Figure 2:**
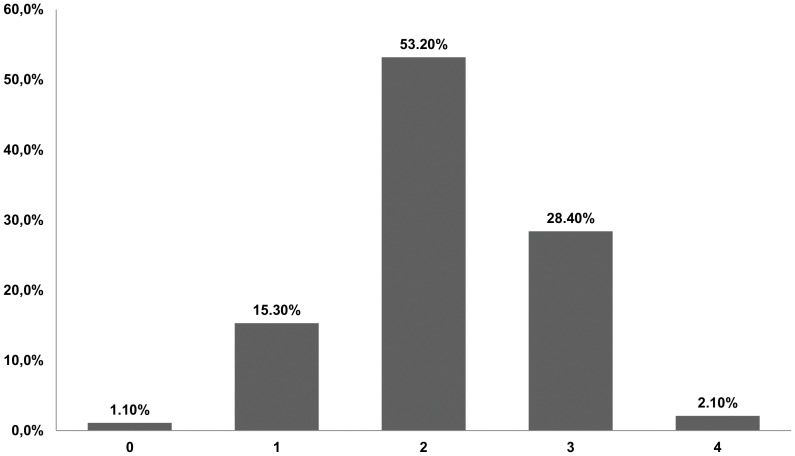
Frequency of systemic inflammatory response signs on intensive care unit admission.

Upon ICU admission, 16.4% of the patients had ≤1 SIRS criterion (Figure 2). The most common SIRS criteria were tachypnea (74.7%) and tachycardia (67.9%). Two or more organ failures were present at admission in 61.5% of patients. Respiratory and hematological dysfunctions occurred more frequently in the non-survivors. Fifty-three patients (27.9%) experienced septic shock within the first 24 hours of ICU admission; however, 96 (50.5%) patients experienced septic shock during their ICU stays. The time for severe sepsis diagnosis was longer in the non-survivors. The patients who developed sepsis in the ward had worse outcomes than those patients in the emergency room (Table 2). The compliance rate with each component of the 6-hour bundle is shown in Table 2. The compliance rate for fluid administration (20 ml/kg crystalloid for hypotension or lactate ≥36 mg/dl) was higher among the survivors.

The clinical and biological variables at the ICU admission and during the ICU stay are shown in Table 3. In the univariate analysis, most of the variables were significantly different between the survivors and non-survivors. Note that more positive fluid balance at 72 hours was also associated with hospital mortality; however this variable was not included in multiple logistic regression due to missing data.

The basal creatinine values (before severe sepsis) were 1.93±1.44 mg/dL. Four patients were under dialysis before ICU admission because of acute Kidney dysfunction. Seventy seven (40.5%) had renal dysfunction (increased serum creatinine > twice the baseline value). Staging AKI according KDIGO, 48 (25.3%) patients reaching Stage 1, 27 (14.2%) a Stage 2, and 94 (49.5%) a Stage 3. There was a strong association between acute kidney injury stage 3 and hospital mortality (Table 4). During ICU stay, 77 (40.5%) patients underwent dialysis (conventional hemodialysis or sustained low-efficiency dialysis). The need for dialysis was higher among non-survivors (75.3% vs. 18.8%, p<0.001). The need for dialysis was not included in our multivariate analysis because of its collinearity with AKI stage 3.

**Table 4:** Severity scores at the ICU admission and the events during ICU stay.

	All patients (N = 190)	Survivors (N = 117)	Non survivors (N = 73)	p value
SOFA admission	5 (4–8)	5 (4–7)	6 (4–9)	**<0.0001**
SOFA at 24 h	5 (4–8)	4 (3–6)	7 (5–11)	**<0.0001**
SOFA at 72 h	5 (3–8)	4 (2–5)	8 (5–11)	**<0.0001**
Delta SOFA 24 h	0 (−1–1)	0 (−1–0)	1(0–3)	**<0.001**
Delta SOFA 72 h	−0.5 (−2–1)	−1 (−2–0)	2(−0.7–4)	**<0.001**
Lactate at admission (mg/dl)	10 (7–16)	10 (6–16)	10 (7–18)	**0.670**
Lactate at 6–12 h (mg/dl)	10 (7–16)	9 (6–13)	12 (8–25)	**0.001**
Lactate at 24 h (mg/dl)	8 (6–14)	8 (6–10)	13 (7–31)	**<0.0001**
Delta lactate 6–12 h (mg/dl)	1 (−3–4)	0 (−5–2)	4 (0–8)	**<0.001**
Delta lactate 24 h (mg/dl)	0 (−4–3)	−2 (−6–1)	3 (0–13)	**<0.001**
APACHE II score	20 (16–23)	18 (15–22)	21 (18–24)	**0.004**
Septic shock	53 (27.9)	24 (20.5)	29 (39.7)	**0.004**
Shock after 24 h	96 (50.5)	29 (24.7)	67 (91.7)	**0.004**
Mechanical ventilation	90 (47.4)	25 (21.3)	65 (89.0)	**<0.0001**
Hemodialysis	77 (40.5)	22 (18.8)	55 (75.3)	**<0.001**
AKI classification				**<0.0001**
Stage <3	96 (50.5)	80 (68.4)	16 (21.9)	
Stage 3	94 (49.5)	37 (31.6)	57 (78.1)	
Reinfection in ICU	34 (17.9)	18 (15.3)	16 (21.9)	0.200
Cumulative fluid balance				
First 6 h after severe sepsis^a^	500 (0–1500)	610 (0–1500)	250 (0–1500)	0.080
First 12 h after severe sepsis^b^	1500 (510–2640)	1500 (565–2569)	1175 (385–2736)	0.350
First 72 h after severe sepsis^c^	4634 (3192–6959)	4301 (3163–6208)	6099 (3657–8391)	**0.007**
First 6 h after septic shock^d^	1500 (774–2069)	1500 (790–2000)	1608 (750–2678)	0.710
First 12 h after septic shock^e^	2190 (1609–3231)	2000 (1394–3036)	2428 (1820–3330)	0.350
First 72 h after septic shock^f^	6928 (4598–8926)	5460 (2096–7117)	8750 (6928–13162)	**0.001**

SOFA: Sequential Organ Failure Assessment score, APACHE II: Acute Physiological and Chronic Health Evaluation II score, AKI: acute kidney injury (from KDIGO), ICU: intensive care unit.

a) n = 189,

b) n = 188,

c) n = 153,

d) n = 50,

e) n = 46,

f) n = 34. Results are expressed as number (%) or median (IQR: 25%–75%). Chi-squared test and Mann Whitney U-test (univariate analysis).

**Figure 3:**
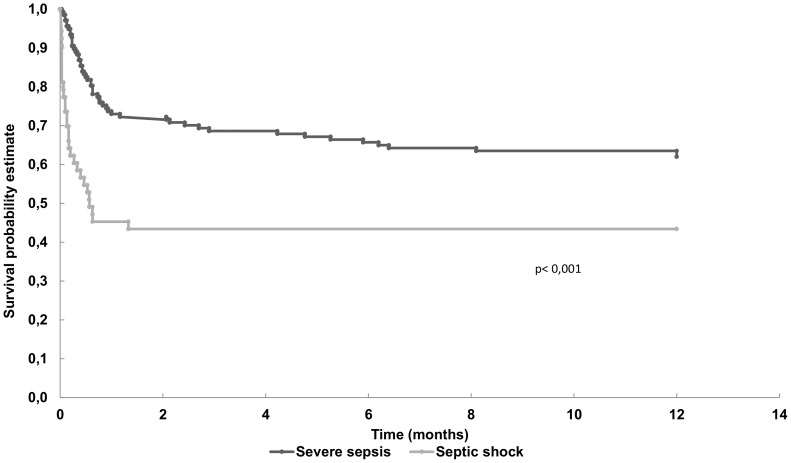
Kaplan-Meier curves (severe sepsis and septic shock) for one year survival after admission to the intensive care unit.

The overall hospital mortality rate was 38.4% (32.1% in severe sepsis patients and 54.7% in patients with septic shock in the first 24 hrs of ICU admission). In the multivariate analysis, the independent determinants of hospital mortality were male gender, delta SOFA score 24 h, mechanical ventilation, hematological dysfunction, admission from ward and AKI stage 3 (Table 5). We could assess the one-year mortality data in all patients and the rate was 42.6% (37.2% for severe sepsis and 56.6% for septic shock). Figure 3 shows the Kaplan-Meier curves for one-year survival after ICU admission.

**Table 5:** Multivariate logistic regression analysis in septic transplant patients with hospital mortality as dependent factor.

	OR (95% CI)	p value
Male gender	5.9 (1.7–19.6)	0.004
Delta SOFA 24 h (per point increase)	1.7 (1.2–2.3)	0.001
Mechanical ventilation	30.0 (8.8–102.2)	<0.0001
Hematological dysfunction	6.8 (2.0–22.6)	0.002
Sepsis admitted from ward	3.4 (1.2–9.7)	0.020
AKI stage 3	5.7 (1.9–16.6)	0.002

OR: odds ratio, CI: confidence interval, SOFA: Sequential Organ Failure Assessment, AKI stage 3: acute kidney injury stage 3.

## Discussion

In our study, we were able to show that the independent risk factors for hospital mortality in renal transplant recipients with severe sepsis and septic shock admitted to ICU did not include the transplant characteristics. There was a lower incidence of SIRS criteria than previously described in other sepsis studies, and there was a higher frequency of opportunistic pathogens causing severe sepsis. We also demonstrated a low increment in the mortality rate one-year after discharge.

The hospital mortality rate for ICU renal transplant recipients varies greatly in the literature, and no study has specifically evaluated septic patients [23]–[29]. Old Brazilian sepsis data from private and public ICU have shown higher mortality rates than in the present study [21], [30]. More recent data still shows a higher mortality rate in Brazil [5] than that reported in some studies conducted in developed countries [2]–[4]. There are some possible explanations for this worst performance. In emerging countries, there are roughly enough resources but there is still limitation in access of care both in private and in public health systems. Sepsis awareness among lay people is restricted which contributes to a delay in searching for care. The gap between scientific evidence and bedside and staff's lack of knowledge, a frequent challenge even in the developed nations, is probably deeper in such settings. Our better findings might be partially explained by a shorter time to sepsis diagnosis [21], which was also associated with survival in our univariate analysis. In addition, the early management of these patients, as assessed by the compliance to Surviving Sepsis Campaign 6-hours bundle [17], [18], was higher than those previously described [21], [22]. The importance of high compliance with the resuscitation bundle to reduce mortality rate was demonstrated in Brazilian private hospitals [7]. In our study, there was a significant lower compliance to fluid administration in non-survivors. Interesting, non-survivors had higher fluid balance at 72 h. This finding suggests that fluids may be essential in the earliest phases of treatment, but late administration may be harmful.

Previous sepsis cohort studies have shown an increment in the mortality rate for sepsis patients (from 7% to 43%) 12 months after the initial assessment (hospital or 28-days mortality) [31]. In our study, no relevant increase in the 12-month mortality rate was observed compared to the in-hospital mortality rate (42.6% and 38.4%, respectively). This interesting and previously unreported finding might be explained, at least partially, by the fact that our patients were younger than those in other sepsis cohort studies [1], [21]. Moreover, they were under continuous surveillance in a transplant center with adequate care during the entire follow-up period.

Considerable variations were present in our findings compared to other sepsis epidemiological studies. Our patients had fewer SIRS criteria. In a cohort, multicenter, observational study in European countries, Sprung *et al.* reported that approximately 90% of their septic patients had ≥3 SIRS criteria, while in our study only 30% of patients had ≥3 SIRS criteria [32]. Moreover, we found that 16.4% of the patients had ≤1 SIRS criteria. This profile of systemic inflammatory response has been previously suggested [19], [33]. Sawyer *et al.* demonstrated that immunosuppressed solid organ transplant patients had significantly lower maximum temperatures and white blood cells counts compared to non-transplant patients [33]. These findings should be taken into account in sepsis studies involving transplant patients, as the need for meeting SIRS criteria to define sepsis could be flawed and may not adequately reflect the actual incidence of sepsis. In fact, the current SIRS criteria to define the presence of sepsis has been criticized even in immunocompetent patients [34].

In our study, the lung was the most common site of infection, which is in alignment with other sepsis cohort studies [22], [35], [36]. This finding was expected, as respiratory infection is the leading cause of ICU admission and acute respiratory failure in renal transplant recipients [23], [26], [29], [37]. The second major source of sepsis was the urinary tract. Although this is the most common infectious complication after renal transplantation [38]–[40], urinary infection might not lead to severe sepsis as frequently as pneumonia even in these immunosuppressed patients. Interestingly, while the data may not be significant, urinary tract infection seems to be associated with lower mortality rates, as previously showed in immunocompetent patients [41]. We also found a higher frequency of microbiologically documented infection by opportunistic pathogens compared with non-transplant patients [1], [8]. This finding was also expected, as infections caused by opportunistic pathogens in solid organ transplant recipient are frequent [42]. However, admissions for severe sepsis did not occur during periods of intensified immunosuppression (in the first months after transplantation or after treatment for acute rejection).

Our analysis showed that the classical factors usually associated with morbidity in this population, such as immunosuppressive regimens, previous rejection treatment and CMV disease, had no prognostic value. Although delayed graft function was associated with mortality, it did not remain in our final multivariate logistic regression model. The only other variable associated with mortality in the univariate analysis, expanded criteria donor, could not be included in the model as it was assessed only in the subgroup that received a deceased-donor kidney. This result aligns with other studies in critically ill renal transplant patients requiring ICU treatment [23], [25], [26], [29].

Delta SOFA after 24 hours of ICU admission, the need of mechanical ventilation, the presence of hematologic dysfunction and admission from the ward and not from the emergency department were previously described as mortality risk factors in critically ill general septic patients [21], [22], [35], [43]–[45]. The most controversial risk factor found in our study was male gender. Clinical sepsis studies evaluating gender-mortality relationships are inconsistent [46]–[49]. Recent studies have suggested that although the incidence of sepsis is greater in men, in-hospital mortality is significantly higher among women [48], [49]. It is possible that gender influences outcomes differently in renal transplant patients. An example of these possible interactions is the reports that grafts from male donors show a trend towards better five-year survival compared to grafts from female donors [50]. Moreover, we did not have data about hormonal concentrations. The complexity of influencing factors did not allow us to evaluate the possible pathophysiological reasons for our finding.

The degree of renal allograft dysfunction during ICU stay was also associated with hospital mortality. As there is no validated classification for AKI in renal transplant recipients, we used a KDIGO definition during the ICU stays [20]. Our results demonstrated that changes in graft function are important and associated with significant changes in outcomes. This result aligns with studies using RIFLE/AKIN definitions in which a worse RIFLE or AKIN class is associated with higher mortality and longer ICU or hospital stay [20]. This study is the first in renal transplant recipients to use a new approach of AKI classification to associate the degree of renal dysfunction with mortality. A previously reported by Nakamura *et al*., higher acute kidney injury states correlate with lower graft survival rates. However, the authors did not present mortality as an outcome [51].

Our study had strengths and limitations. We included a homogeneous population of renal transplant recipients in a consecutive fashion. We assessed several transplant and sepsis characteristics, including treatment adequacy, which could interfere with patient outcomes. In addition, we used a new AKI classification approach. These contributions are relevant considering the paucity of data currently available in the literature. The study also has some limitations, the most important being the retrospective nature of our data collection. Second, our study has a single-center design, which limits the reproducibility of our findings. Third, we did not have a control group with septic non-transplanted patients and transplanted patients without sepsis. Fourth, we limited our analysis to ICU patients and did not include patients with severe sepsis in other hospital settings. The relevance of this limitation should have been minimized because in this institution, the vast majority of the septic patients are admitted to the ICU. Fifth, a better characterization of AKI is lacking. We do not have data regarding estimated glomerular filtration rate, time for dialysis onset or its duration, and long-term graft function. Moreover, we did not assess the role that acute rejection could have played in graft dysfunction. We also only consider creatinine and not diuresis in our AKI classification, which may have underestimated the number of patients with late stage diseases. However, controversy exists regarding the impact of this assessment in the score ability to predict prognosis [52]. Sixth, we have no data regarding adrenal insufficiency in our study. Besides the possibility of corticosteroid insufficiency related to critical illness or sepsis, previous chronic use of prednisone in nearly all patients could suppress the hypothalamic-pituitary-adrenal axis (HPA). This was the main reason for hydrocortisone administration. Another reason was the need for immunosuppressant drugs to prevent rejection, since all other immunosuppressant agents were discontinued at ICU admission.

## Conclusion

Hospital mortality in renal transplant patients with severe sepsis and septic shock was associated with male gender, admission from the wards, worse SOFA scores on the first day and the presence of hematologic dysfunction, mechanical ventilation or advanced graft dysfunction. Transplant-related variables had no prognostic value.
